# Home Away from Home: How Undergraduate and Graduate Students Experience Space and Place in a new Health Sciences Building

**DOI:** 10.1177/08445621231190581

**Published:** 2023-08-01

**Authors:** Karen LeGrow, Sherry Espin, Lois Chui, Don Rose, Richard Meldrum, Mary Sharpe, Enza Gucciardi

**Affiliations:** 1 Daphne Cockwell School of Nursing, Faculty of Community Services, Toronto Metropolitan University, Toronto, ON, Canada; 2 McMaster Children’s Hospital, Faculty of Community Services, Toronto Metropolitan University, Toronto, ON, Canada; 3 School of Occupational and Public Health, Faculty of Community Services, Toronto Metropolitan University, Toronto, ON, Canada; 4 School of Midwifery, Faculty of Community Services, Toronto Metropolitan University, Toronto, ON, Canada; 5 School of Nutrition, Faculty of Community Services, Toronto Metropolitan University, Toronto, ON, Canada

**Keywords:** higher education, space and place, learning environments

## Abstract

Buildings contribute in crucial ways to how students experience learning spaces. Four schools within a faculty (nursing, nutrition, occupational and public health, and midwifery) moved into a new Health Sciences building Fall of 2019. This new building created a unique opportunity to explore the intersection between higher education and learning space design, informed by concepts of space and place, and students’ profession specific and interprofessional learning experiences in a new Health Sciences building. A qualitative descriptive design was used. All undergraduate and graduate students within the four schools were invited to participate. Focus groups were undertaken to gain a rich understanding of students’ experiences and views of their space and place of learning. Data collection involved focus group data from profession specific participant users and interprofessional participant users. Inductive thematic analysis of focus group transcripts generated an initial coding scheme, key themes, and data patterns. Codes were sorted into categories and then organized into meaningful clusters. A building planning development project document relating to the vision, intentions, design, and planning for the new building provided content from which to view the study findings. The study data contributed to the conversation about space and place and its influence on higher learning within specific intraprofessional and interprofessional student groups and provided insight into the process of actualizing a vision for a new learning space and the resultant experiences and perceptions of students within that space/place.

## Background

Evidence suggests that the interactions between students in higher education and their learning environments is a key component towards enhancing student experiences (Beckers et al., 2016; Castilla et al., 2017; Cox, 2018; Hawick et al., 2018, 2020; Leijon, 2016; Matthews et al., 2011; Mthimunye & Daniels, 2019). The connection between space, place and interprofessional learning can create professional cultures and identities through the hidden curriculum (Nordquist et al., 2011). Further, interpersonal relationships outside of the classroom have an impact on professionalization, socialization, and enculturalization of students into their health and social care professions (Hafferty & Castellani, 1998). Space and place, as sensitizing concepts, has received increasing attention in higher education (Temple, 2008). These concepts are used to better understand how learning environments shape student experiences and the development of university communities within higher education (Ellis & Goodyear, 2016; Leijon et al., 2022; Temple, 2008)

## Literature review

Current literature suggests that academic spaces and places play an integral role in higher education. Learning space design, informal learning spaces, and places of higher learning have emerged as important elements influencing student learning experiences.

Classroom layout (Beckers et al., 2016; Castilla et al., 2017), and spatial design (Matthews et al., 2011) can impact student experience and learning. In a classroom environment, student experiences are affected by functionality and layout, the perceived comfort in the space, the ability to concentrate, and artificial lighting (Castilla et al., 2017). Light is found to be important in helping students not feel suffocated in a space (Cox, 2018). Further, the design of learning spaces may portray connotations for how the space's purpose is intended to be used (Beckers et al., 2016; Leijon, 2016). Physical features in a space may contribute to academic and social behaviours that are impacted by the spatial design and the perceived noise levels associated with the space and associated interactions with others in the space (Matthews et al., 2011).

Informal learning spaces may support student interactions outside of the traditional classroom (Matthews et al., 2011). Beckers et al. (2016) directs our attention to how students share social spaces with one another, and how intra-professional and interprofessional interactions in informal spaces can be considered real learning. Further, there is an emphasis on the perceived effectiveness of space. Students value informal learning spaces that promote effectiveness, usability, and comfort (Beckers et al., 2016; Matthews et al., 2011). Informal learning can portray a sense of socialization, and a sense of urgency through pressured silence in a communal space particularly when there are deadlines approaching (Cox, 2018).

Learning environments may promote a sense of belonging for students whereby social networks are created (Matthews et al., 2011). Further, environments have the power to shape our sense of who we are and what we do (Kuntz et al., 2012). After moving to a new faculty building, graduate students found that interactions and perceived sense of community between students lessened, as their learning spaces were impacted by spatial configurations (Kuntz et al., 2012). Interactions among students are affected by perceived flexibility to move around in the space (Leijon, 2016). Furthermore, space and place can also impact our sense of interpersonal meanings (Leijon, 2016). For example, power relations may be portrayed in a lecture hall where the teacher encompasses the power at the centre of the room, whereas in a group room, the power may be perceived as more leveled and portray a sense of equality between students and teacher (Leijon, 2016).

In summary, current research demonstrates the importance of understanding space and place and its influence on student learning experiences (Beckers et al., 2016; Brewer et al., 2017; de Jesus et al., 2014; Konings et al., 2005; Kuntz et al., 2012; Matthews et al., 2011; Mthimunye & Daniels, 2019; Neil & Etheridge, 2008) and academic outcomes (Brooks, 2011; Espey, 2008). Informal learning spaces, students’ interactions within a space, and a sense of place play impactful roles in higher education contexts within and between professions. Therefore, a need to further explore student experiences of space and place in an intra-professional and interprofessional context is warranted to examine the interplay between space and place, student connectedness, and professional identity. Thus, the aim of this study is to examine how a newly constructed health sciences building has shaped student experiences and professional development for students within and between interprofessional programs.

## Theoretical orientation

Recognizing the importance of the conceptual and practical relationship between space, place, and learning in interprofessional education is central to constructing learning spaces that provide opportunities for students from different professions to collaborate, communicate, and interact to construct places of learning (Nordquist et al., 2011). We understand profession learning as intra-professional specific learning, i.e., students from the same professional background learning together. Whereas interprofessional learning (education) occurs where students from two or more professions learn about, from, and with each other to enable effective collaboration and improve health outcomes (WHO, 2010). We propose that space and place are two sensitizing/critical concepts that provide a frame from which to understand student learning experiences within higher education. For this study, space is defined by geographical location and material form – referring to abstract geometrics (Gieryn, 2000); place is defined not only by geographical location and material form, but by the meaning and value that people associate, attach, and invest in a physical space (Poland et al., 2005). More specifically, space and place can be used to explore relationships, communication, and processes of learning. For example, how space is used for learning, how place can affect the opportunities for learning and types of learning, and how together space and place influence our understanding of learning, education and interprofessionalism (Kitto et al., 2013).

## Purpose

To explore the intersection between higher education and learning space design, informed by concepts of space and place, and students’ profession specific intraprofessional and interprofessional learning experiences in a new Health Sciences building.

## Research design

A qualitative descriptive study design (Bradshaw et al., 2017; Green & Thorogood, 2018; Sandelowski, 2000) using a thematic analysis approach was used to examine how a new health science building shapes student experiences and professional development. Focus groups were undertaken to gain a rich understanding of students’ experiences and views of their space and place of learning.

## Method

### Setting

This study took place in a Health Sciences Building on the campus of a post secondary university. The University is situated in a large diverse urban city and offers over 125 undergraduate and graduate programs, has a student population of over 48,000, and has partnered with 176 institutions in 49 countries.

The Building Development Project began in June 2011, the schematic design was completed January 2014, and the building opened for the beginning of the 2019–2020 academic year. Components of the building's vision statement included key themes such as identity, experience, legacy, inclusivity, student experience, sustainability, pedagogy, and civic identity (xxxxxxx, 2014). Key structural features included: 1) teaching, administration, research facilities for 4 academic Health Sciences Programs, 2) 6 classrooms, ranging from 60 to 225 seats, 3) significant food services program (e.g., Marche-style commercial kitchen and dining seats for 80 people), 4) fabrication zone dedicated to rapid prototyping, 5) student residence with at least 250 beds, and 6) below-grade parking. The information contained in the planning development project document relating to the vision, intentions, design and planning for the new building provide a context from which to view the study findings.

### Ethics

Prior to recruitment and data collection activities being undertaken, ethics approval was obtained from the university's REB to conduct the study.

### Recruitment

All undergraduate and graduate students within the School of Nursing, the School of Nutrition, School of Occupational & Public Health, and the School of Midwifery at the University were invited to participate. Students received invitations to participate in the study via email from their respective program administrations. Three separate sequential emails were sent to students by program administrators to enhance recruitment efforts. Invitations provided a brief description of the study, expectations of participants, and an opportunity to have questions answered by the study research assistant (RA). If interested, students contacted the RA, they then proceeded with the consent process and obtained a signed consent form. Participants received an honorarium upon completing the focus group as an appreciation of their time.

### Sample

A total of 27 students participated in the study. The students represented undergraduate students from nutrition (n  =  4), occupational and public health (n  =  6), midwifery (n  =  4) and nursing (n  =  8) in years one to four of their respective programs, and graduate nursing students (n  =  5) from the Master of Nursing program in the second year of their program (Table 1).

**Table 1.** Study participants.

Program of Study	Student participants
Nursing (Undergraduate students)	n = 8 year 2 (n = 2) year 3 (n = 3) year 4 (n = 3)
Nursing (Graduate students)	n = 5 year 2 (n = 5)
Occupational and Public Health (undergraduate students)	n = 6 year 2 (n = 3) year 3 (n = 1) year 4 (n = 2)
Midwifery (undergraduate students)	n = 4 year 3 (n = 3) year 4 (n = 1)
Nutrition (undergraduate students)	n = 4 year 2 (n = 1) year 3 (n = 2) year 4 (n = 1)

### Data collection

Data collection took place during the initial phase of the COVID-19 pandemic when students engaged exclusively in online learning activities, therefore data collection only involved their perceptions of the space during the Sept. 2019-March 2020 academic period. A total of six focus groups were conducted over the course of the study. Three focus groups were conducted during the Fall 2020 semester, one conducted during the Winter 2021 semester, and two conducted during the Spring 2021 semester. Each focus group was coordinated and scheduled by the RA with interested participants based on their availability. Participants were provided with a link, unique to their assigned focus group, to access the digital platform where the focus group would be taking place. For this study, the Zoom platform was used to conduct all focus groups, with the security feature activated to ensure a safe space for the activity. A majority of the focus groups were profession specific (n  =  5/6), with one interprofessional focus group including participants from four profession specific programs (n  =  1/6), (nursing, occupational and public health, midwifery, and nutrition). Focus groups ranged from a minimum of 25 min to a maximum of 60 min. Focus group question are outlined in Table 2.

**Table 2.** Focus group questions.

Focus Group Questions: 1) What are your experiences of being in the Health Science building? 2) How are you using the space? 3) What does the building offer in terms of possible collaboration, connections, and inclusivity? 4) How has the space shaped your learning experiences? 5) Is there anything further that you would like to share?

### Data analysis

Focus group transcripts were inductively reviewed using thematic analysis (Sandelowski, 2000, 2010) to generate an initial coding scheme, key themes, and data patterns. Codes were then sorted into categories and then organized into meaningful clusters. Consistent with this approach, all members of the research team were immersed in the focus group data via in-depth reading and coding of the transcripts. Coded responses were collected and used to construct a series of codebooks. The research team met at regular intervals to discuss the organization of the codebooks, which led to the construction of the themes. Several strategies were used to enhance the rigor or trustworthiness (Lincoln & Guba, 1985; Schwandt et al., 2007). We engaged in the 1) credibility through interprofessional triangulation of research team members and study participants, 2) transferability with purposeful sampling of study participants and a rich description of the study findings 3) dependability with the creation of an audit trail to demonstrate the research process and 4) confirmability through practicing reflexivity where the team engaged in individual and team reflections (Lincoln & Guba, 1985).

## Findings

**Figure 1.**
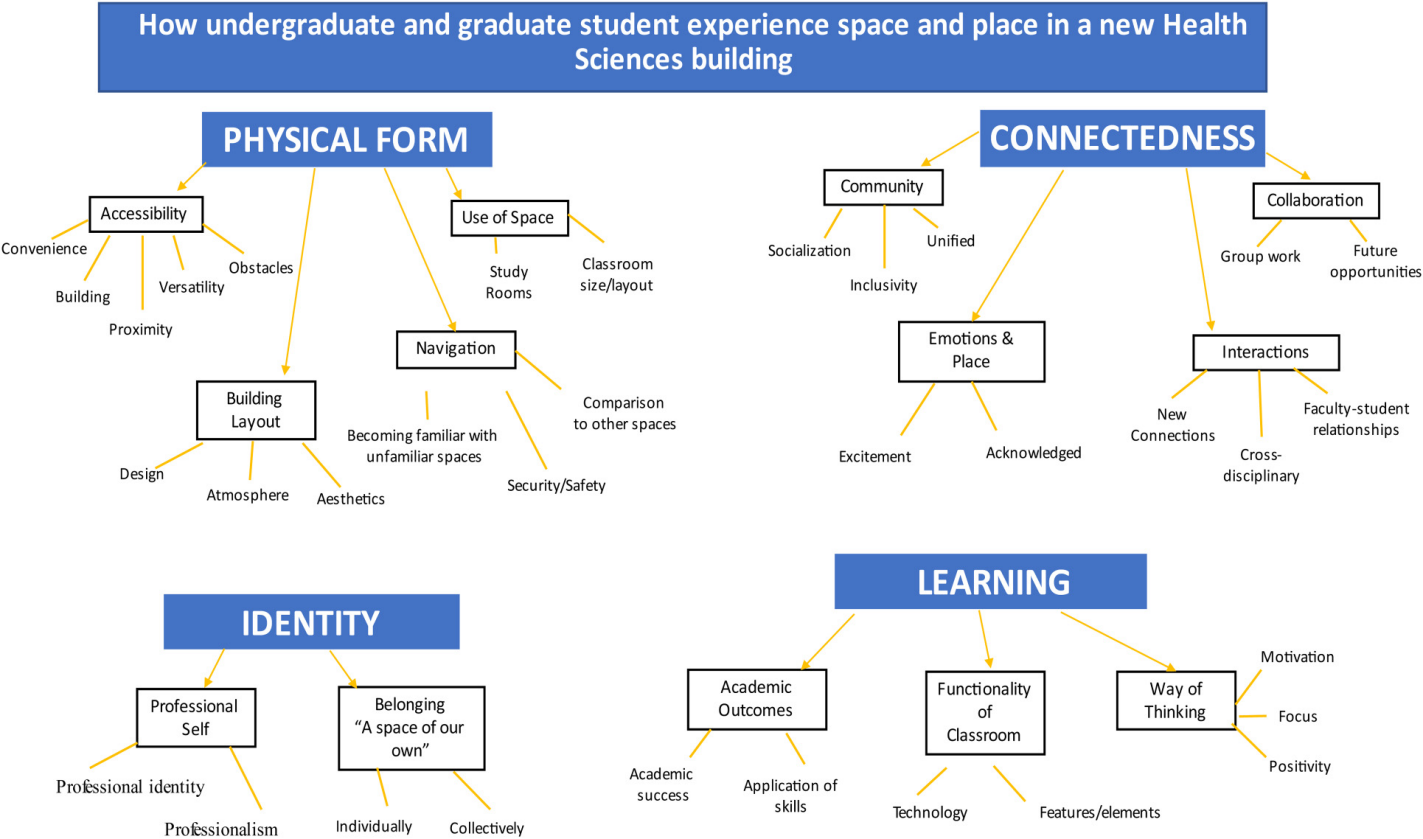
Coding tree.

Focus group data revealed how space and place influenced undergraduate and graduate student learning experiences in the new Health Science building. Key themes that emerged included 1) physical form, 2) connectedness, 3) identity, and 4) learning, which highlighted different aspects of how students give meaning to a space and thus create a place. Each key theme and their respective sub-themes are identified (Figure 1) and described with student statement/quotes.

### Theme 1: physical form (space)

Students identified that the building's physical form such as accessibility features, layout, and use of and navigation within the space as significant to their experience within the new Health Sciences building. Accessibility to the building and its functionality was described as the availability of gender-neutral bathrooms, chest/breastmilk pumping room, and wheelchair accessibility. Elements of the space were deemed as convenient and versatile, and thus enhanced student informal and formal learning experiences within the building. For example, versatility was described as the flexibility to make a space one's own by moving furniture around, and the variety of space accessible to students. One student enjoyed that they were “able to spend part of the day in one room and then move to another place and giving your work structure be in alignment with the space. I would use the graduate room for when I needed to edit and do mentally demanding work. And if it was something that I could devote a little less energy on, I would use something like the comfy chairs” (07NG).

Proximity was described as the building's location in relation to the city's transit system and to other student buildings. “[The health sciences building] is not a huge school, but a lot of our buildings are in random places, so having something that was straight forward was nice that it wasn’t hard to find” (04OH), a student shared when comparing the old to the new health sciences building. Physical form also encompassed the building's layout. For students, building layout referred to the building's aesthetic or physical appearance that focused on design and atmosphere. Areas of the design highlighted by students included its open concept and use of large windows, inviting spaces/sense of invitation, and reflecting a modern design. One student highlighted that the building was “pristine and clean, inviting…It made me feel in a way, some aesthetics around the program, overall, it's a perception of the program as well” (02OH). Atmosphere pertained to the cleanliness and light/brightness in the space and its impact, including feeling stimulated, awake and relaxed. More opportunities for greenspace in the building was mentioned as a consideration.

Obstacles pertaining to accessibility emerged from the focus groups. Obstacles were identified by students as perceived hindrances to accessibility within its physical form. Examples included difficulty accessing study rooms, accessibility to parking, length of wait time for elevators, and the number of available washrooms. The use and navigation of the space included the flexibility and accessibility of space, namely study rooms in the building. Classroom layout was described as a factor that affected learners as classroom space was described as being small in size and reported feeling cramped. As the space was unfamiliar to students, there was a transition from unfamiliarity to familiarity of the space. When comparing their experience of being in the previous health sciences building to the new one, one student reported “it was neat, I felt like a guest in that [midwifery] space, but it sort of became more comfortable over time” (06NG). For another student that had more classes in the new health sciences building, they “were able to access other parts of the building and have a chance to explore the building more” (02OH). Students felt both positive and negative emotions associated with a security presence. The presence of feeling safe within the building was connected to the security personnel present however, lack of security emerged for students when considering the geographical location of the building, as it is centrally located in a large urban city.

### Theme 2: Connectedness (place)

Connectedness speaks to the interactions, collaboration, emotions, and sense of community evoked within the building space. The presence of lockers, lecture halls, and layout of furniture in informal learning spaces within the building were physical structures that promoted student connectedness. Further, community emerged through a perceived sense of unity, socialization, and inclusivity. One student shared, “the [building] provides a space for all of us to be together as a community. It's a great space that helps me feel united with my program/school, a part of our own busy beehive within the [University]. By being all together in the space, including students, staff, faculty, I become more familiar with the faces” (08NG).

Being situated in the same building as students and faculty of other professions promoted unity as described by the participants. For some, being in the same space as future healthcare team members was described as home. “I felt like I was around the healthcare team which really helped. Of course, like the future healthcare team and I felt like I found I had a home in that building” (14NG). One student described the presence of the lockers giving them a feeling of “home away from home” (MW01), a feeling in which they described once they moved into the new building. Students perceived the physical structures of the building as promoting a sense of inclusivity. This idea for example, was suggested with the circular couches throughout the building.

Connectedness emerged in the different forms of interactions noted by students, including new connections, cross-professional interactions, and faculty-student relationships that helped create new opportunities for students. “I was able to talk to different students in the program and I learned that you know, it doesn’t matter you don’t have to ask people from the same program about the question, you can ask other people too, they might know as well, because we’re all in the same building. I found it very helpful” (06OH). For another student, “I appreciated that it allowed me to make connections with other students in the program. I was really anxious about going back to school after a really long time about meeting new people, and becoming integrated into the program really helped facilitate connections and helped me form friendships, like having that space and that place to be together” (04MW).

Collaboration was described as the physical space that allowed for opportunities for students to work in groups (i.e., movable chairs, big tables, open spaces, lounges, and study rooms). Positive emotions described, included a sense of excitement with moving into the new building and a sense of feeling acknowledged. One student felt that “ the whole entire environment demeanor of it was extremely positive and having the glass windows open and the sunlight…it just made it even more calming and a better environment, more secure environment where I could study and relax and feel better about myself” (03NT). Another student noted that “the space made me feel acknowledged and appreciated, just seeing a space that was made for students” (03MW).

Excitement was experienced when thinking of the future collaboration opportunities with other faculties and students. There was also a sense of excitement when walking into the building. Lastly, students felt a sense of acknowledgement from the university that their programs were in a new building. In contrast to not feeling acknowledged in their earlier learning experiences that took place in buildings on campus with limited facilities. Interestingly, students noted future opportunities for further collaboration that could take place in the building, including potential events, and furthering the use of the building for interprofessional collaboration and conferences.

### Theme 3: Identity (place)

Identity reflects the individual professional self and collective professional identity that is embodied when individuals are in the building. The physical space enabled individuals to embody a sense of professionalism and thereby identify with their respective health professional community. Some students perceived their programs place within this new health science building as enhancing their professional identity. One student expressed that “this space helps me feel that my learning experience was more professional and more taken seriously by those in power; more academic, and more respected…I started to dress more professionally to match the space as a result which affected how I perceived my own sense of belonging with the community” (08NG). Professional identity emerged as students felt more prepared to go into their clinical areas after learning in the building's simulation lab space. Other students felt a sense of belonging knowing that their program was located within the same building as other health profession programs. One student noted, “I look at programs like Nursing and Midwifery as premium programs…I am not gonna lie I definitely had that perception, and I would say the space, putting me in the same building, I’m attending my classes in the same building as all these other programs…That made me feel more connected, and a part of the larger health sciences umbrella” (02OH).

### Theme 4: Learning (space)

Learning speaks to how the space is perceived to effect student learning. Students attributed classroom layout, lab space, and classroom features to their ability to achieve higher academic success. Specifically, the lab space in the building was described as an opportunity for students to apply theoretical knowledge to skills, helping them consolidate knowledge. “I really enjoyed the sim labs and the experiential learning suites. I really enjoyed learning in those for the clinical skills courses, because it gave us an opportunity to learn in a clinical space without necessarily as much supervision, which made you feel comfortable making your mistakes and making errors in a safe space with friends, while you’re all learning together” (01MW).

Further, classroom space and its encompassing features were viewed as both promoters and barriers to student learning. Promoters included a sound-proofed effect of walls, plugs available to students, and the flexibility to adjust classroom furniture. “I used the services like the study rooms towards the end of the fall term, and I felt like they had whiteboards, some had computers that you can present off of. I found that really benefitted my grades and collaboration with my colleagues” (02NT).

Some barriers included technological difficulties around screens within classrooms, and the lack of space some students feel they had in the class such as no space to put their laptops on desks. “Our professors use the projectors a lot when they were showing their PPTs, but there were often technical difficulties, which I guess happens a lot. But it wasn’t technical difficulties that the profs couldn’t fix themselves” (01NG). Finally, the space was perceived to effect students’ attitudes towards learning, including the feeling of motivation, the ability to remain focused, and a sense of positivity. “I could study better. I would have a more positive attitude towards studying, and I thought I’d have a clearer mind when I’m studying and I could focus and overall, that affected my marks. I noticed that correlation between the building and my marks.” (03NT).

## Discussion

We explored how a new Health Sciences building has shaped students’ learning experiences and professional development within and between interprofessional programs. Students’ experiences clustered around common themes of physical form, connectedness, identity, and learning. The building's guiding principles document provided an organizing structure to elucidate our study results.

One of the building principles was to create an aesthetically beautiful and welcoming space for new communities of learning. Creating a welcoming space for new communities of learning was highlighted by students, as they described the design features of the building including its openness, sense of invitation, and modern look. They expressed that the new building was very welcoming. This was evidenced by feeling welcomed by the openness, the light, and the cleanliness of the new building. These reports align with previous evidence that spatial design and physical features in a space such as building layout and functionality along with lighting can impact learning (Beckers et al., 2016; Castilla et al., 2017; Cox, 2018; Matthews et al., 2011). However, students noted barriers related to parking, and study spaces. Parking was an issue for the group of mature graduate students as they found the lack of parking spaces available around the building to be a deterring factor from studying on campus. Some students expressed that study rooms were only available at certain times in the day, while others expressed that they had no knowledge that study rooms existed within the building.

Another design principle of the building was to provide inclusive and seamless accessibility. Accessibility included equitable use, flexibility, low physical effort, and size and space for mobility and use. The new building provided gender neutral bathrooms, breastfeeding/pumping room, and wheelchair accessibility. Students in this study spoke to the building's convenience, proximity, versatility, and accessibility. They reported that it was convenient to have faculty/staff offices and informal/formal learning spaces located in one building and close to other campus buildings and city transit. Students also noted the variety of learning spaces available and the flexible use of the space and its furniture. Students described a feeling of inclusivity from the placement of the furniture within the building that promoted student interaction and socialization and the variety of spaces that allowed for different types of work to be undertaken and completed. As suggested by Leijon (2016), interactions among students are impacted by their perceived flexibility to move around in learning spaces. Obstacles noted by students were the perceived hindrances to accessibility in the health sciences building. This included difficulty accessing elevators and washrooms due to lengthy wait times.

The intent of the building's design was to accommodate a full range of teaching/learning models to promote collaborative and transformative learning experiences. Students in this study identified that the building facilitated different learning styles through the various learning spaces (facilities) such as lecture halls, skills and simulation labs, and study rooms. Students noted that the various learning spaces afforded them opportunities to apply theoretical knowledge to practice and consolidate their learning that contributed to their academic success. References by students to different kinds of learning spaces within the health sciences building, align with Nordquist (2016) organizing concept of a ‘learning landscape’, specifically classroom and building, as two landscapes that reside within two larger landscapes, that of a campus and the wider urban fabric (p.63). However, there were reports of barriers to learning noted by students, linked to classroom functionality, where classroom technology malfunctions were noted, and use of space, where the size of the classrooms limited the flexible use of the space.

Lastly, identity was a guiding principle of the building that was designed to be a hub for interprofessional collaboration with celebrating a holistic approach to health and wellness. Many students in the study appreciated the informal interprofessional collaboration and learning, through being in the same space as other schools and engaging in conversations with other students from other professions. Similar to earlier research by Beckers et al. (2016) student interactions within social spaces can be thought of as important learning experiences.

Student participants noted how being in the presence of other schools in the faculty helped shape their identity and connectedness suggesting that ‘place’ has an essential role to play in students personal and professional identities pre-licensure. Promoting a sense of belonging and shaping who they are and what they do through learning environments that provide social connections and networking has been identified as impacting learners’ experiences (Kuntz et al., 2012; Leijon, 2016; Matthews et al., 2011). While students noted the opportunities afforded in the new space, they also noted a lack of formal interprofessional collaboration within the space and recommended informal and formal opportunities to do so in the future.

## Conclusion

In conclusion, undergraduate and graduate students’ formal and informal learning experiences within a newly constructed health sciences building were explored. The concepts of space and place were reflected in student accounts of their learning experiences. Students’ perceptions and reflections noted four key themes: the buildings physical form, spaces that allowed for a sense of connectedness and a formation of professional identity, and learning spaces influence on their academic success. In moving forward, the importance of accessible physical spaces that function as intended and having opportunities for connection and belonging is essential to students’ sense of place and their professional and interprofessional identity. When planning and designing new buildings for professional and interprofessional learning we may consider, how do we create a home away from home for students within the health professions?
